# Improvement of sleep quality 6 months after total knee arthroplasty: a systematic review and meta-analysis

**DOI:** 10.1186/s13018-021-02493-4

**Published:** 2021-05-28

**Authors:** Ali Alipourian, Negin Farhadian, Ehsan Zereshki, Habibollah Khazaie

**Affiliations:** 1 Sleep Disorders Research Center, Health Institute, Kermanshah University of Medical Sciences (KUMS), Kermanshah, Iran; 2 Research Center for Environmental Determinants of Health (RCEDH), School of Public Health, Kermanshah University of Medical Sciences, Kermanshah, Iran

**Keywords:** Meta-analysis, Pittsburgh Sleep Quality Index (PSQI), Total knee arthroplasty (TKA), Systematic review

## Abstract

**Background:**

Total knee arthroplasty (TKA) is an accepted, effective treatment to restore function, relieve pain, and improve the quality of life in patients with advanced osteoarthritis. One complication of this major surgery is impaired sleep quality. This study examines the quality of sleep in patients undergoing TKA before and after their operation.

**Methods:**

All relevant records were obtained using a systematic search in three online databases: PubMed, Scopus, and Cochrane library. Out of the 177 records retrieved, only eight matched the inclusion criteria. Due to the lack of sufficient data, only four studies entered the meta-analysis. Values reported for sleep quality based on the Pittsburgh Sleep Quality Index (PSQI) were extracted from patient records before and after surgery. A random-effect model was used to analyze the data.

**Results:**

The results of the meta-analysis show a significant difference in the improvement of sleep quality after surgery at two time points of 4–6 weeks after surgery from the preoperative baseline (SMD − 0.16; 95% CI − 1.05 to 0.74; P = 0.0) and 3–6 months after surgery from the preoperative baseline (SMD − 0.92; 95% CI − 1.61 to − 0.24; P = 0.0).

**Conclusions:**

The results show that TKA generally improves the patients’ sleep quality. Although some studies reported disrupted sleep quality in periods close to the surgery (especially in the early days after surgery), all studies have reported improved sleep quality in the late postoperative intervals.

## Introduction

Knee osteoarthritis (KOA) is an arthritic disease with a higher prevalence among adults over the age of 65. The risk of developing KOA increases with age [1]. KOA causes a lot of pain, affects individual’s quality of life, reduces their ability to perform daily tasks, and causes depression and anxiety [2, 3]. One of the main symptoms in the advanced stages of KOA is nocturnal knee pain and pain when the individual wants to move [4]. Joint replacement surgery, or total knee arthroplasty (TKA), is one of the main treatments for end-stage KOA.

Arthroplasty is an effective modality for treating patients with osteoarthritis through improving their mobility and quality of life [5, 6]. Recent decades have witnessed an increase in orthopedic surgeries due to the increasing older adult population, a higher prevalence of arthritic diseases, and joint destruction [7, 8]. The need for arthroplasty is expected to increase six-fold in 2030 compared to that of 2005 [9, 10].

Knee arthroplasty is among major surgeries which impair patient’s sleep quality due to the severity of its trauma [6, 11, 12]. Normal sleep is both physiologically and psychologically essential for patient’s rehabilitation process [13]. Sleep disturbances occur in 50% of patients after TKA, and are an important post-op complication that have not yet been properly understood, and sometimes neglected [14, 15]. Persistent post-op sleep disorders not only aggravate the pain but also prolong recovery time and hospital stay, which negatively affect patient-physician relationship [9]. Chronic pain affects sleep quality in that people with chronic pain suffer delayed onset of sleep, more waking hours, disturbed sleep quality, and fewer sleep hours [16–18]. One of the most common complaints of people after surgery is sleep disturbances, which are often associated with severe pain [19]. Interventions that target sleep disorders may accelerate recovery in patients after orthopedic surgeries and other surgical procedures [20].

Although suffering sleep disorders is one of the conditions that indicate the need for arthroplasty, few studies have addressed the issue. Studies have shown that knee arthroplasty can improve patients’ quality of life [15, 21]. Accordingly, some indexes such as the Short Form 36, the Knee Society Score, and the Western Ontario and McMaster Osteoarthritis Index improve after surgery [22, 23].

Studies that report changes in sleep quality after arthroplasty have yielded different results. A study with 110 patients reported sleep disorders one month after surgery due to increased pain during this period as well as limited function 3 months after surgery [20]. Another study with ten patients who underwent arthroplasty found that rapid eye movement (REM) sleep decreased at night immediately after surgery, but returned to the preoperative baseline four nights after surgery [13]. Patients undergoing arthroplasty had a worse sleep quality 1 month after surgery compared to the control group [8]. Fewer sleep disorders and improved sleep quality were also reported 3 months after arthroplasty [24].

Sleep disorders arising after TKA are a complex process and vary greatly from one patient to another. Finding a solution to improve sleep and recovery in these patients requires more information about sleep disorders after surgery. Hence, this study aimed to evaluate the effect of TKA on sleep quality. This study evaluated the sleep quality of patients undergoing TKA at two time points after surgery. To this end, sleep quality data were obtained from reviewed studies based on Pittsburgh Sleep Quality Index (PSQI) and analyzed at a time point close to surgery (4–6 weeks after) and another later time point (3–6 months after).

## Methods

**Fig. 1:**
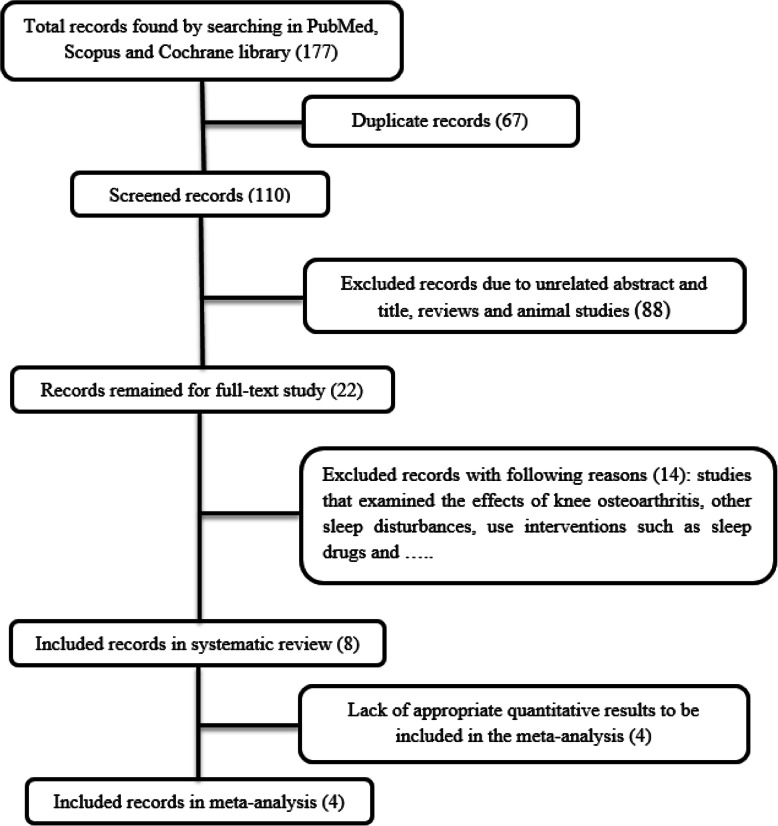
Flow diagram of the systematic searching process

Preferred Reporting Items for Systematic Reviews and Meta-Analyses (PRISMA) checklist was used for this review study [25]. Studies examining the effect of TKA on sleep quality were included. Eligible studies were retrieved through a systematic search of three databases of PubMed, Scopus, and Cochrane library. Keywords used for search include sleep quality, knee, arthroplasty, and replacement. The search strategy to find relevant studies is displayed in Fig. 1. Inclusion criteria included any English-language study that examined the quality of sleep after knee arthroplasty in humans.

Studies that evaluated postoperative sleep disorders such as insomnia or sleep duration, the effect of types of surgical procedures such as fast-track knee arthroplasty, the impact of interventions such as using zolpidem or melatonin on sleep quality, or the quality of sleep in knee osteoarthritis were excluded.

Subjective tools were used to evaluate sleep quality in these studies. Few studies evaluated daytime sleepiness using the Epworth Sleepiness Scale (ESS). Values of sleep quality were extracted from the studies based on the PSQI before and after surgery. The total score of all questions in PSQI is 21 points, and a higher score indicates poorer sleep quality [26]. A total score above 8 indicates poor sleep quality [27]. Continuous average PSQI score with mean and SD was analyzed using mean differences. Heterogeneity among studies was assessed using the I-squared test. The quality of the studies was evaluated independently by two researchers using the Newcastle-Ottawa Scale (NOS). The Begg and Egger tests were used to assess publication bias. All statistical analyses were performed in STATA software.

## Results

**Fig. 2:**
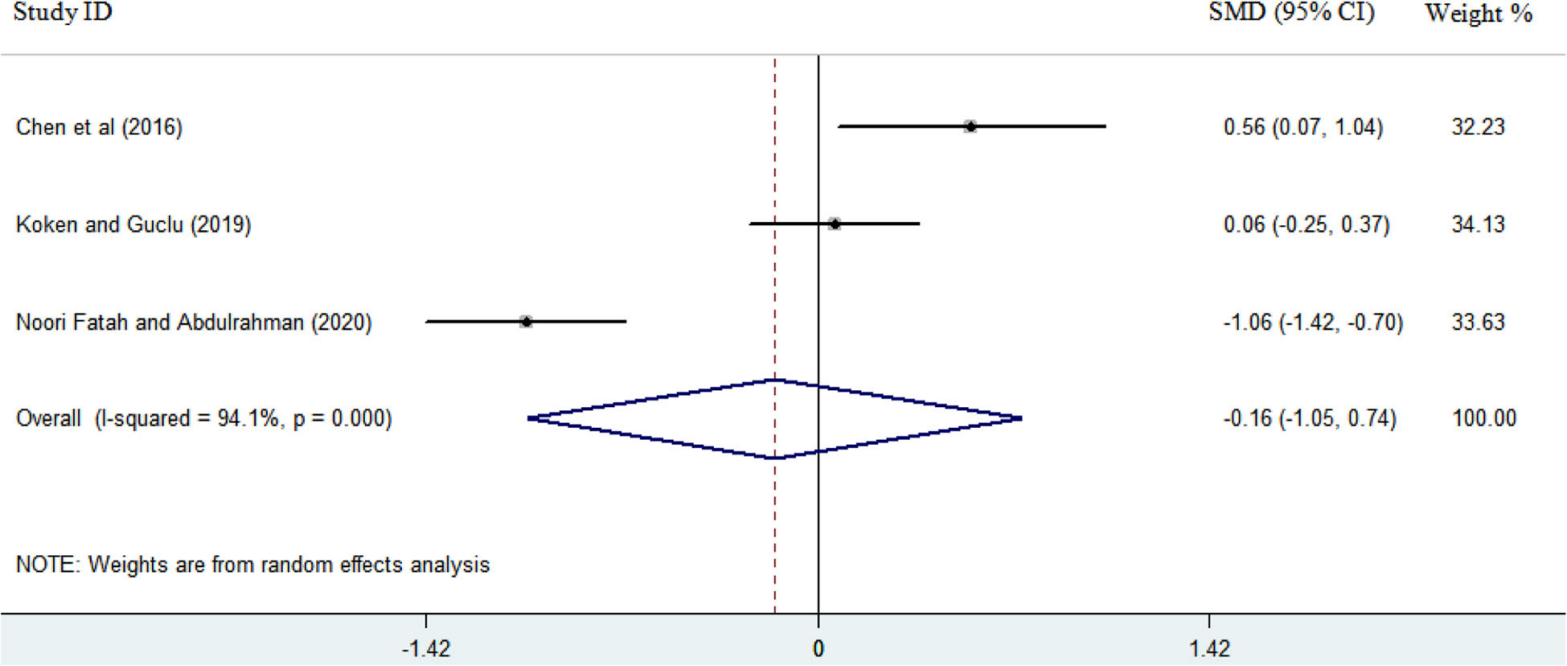
Forest plot showing the effects of TKA on sleep quality at time point of 4–6 weeks after surgery

**Fig. 3:**
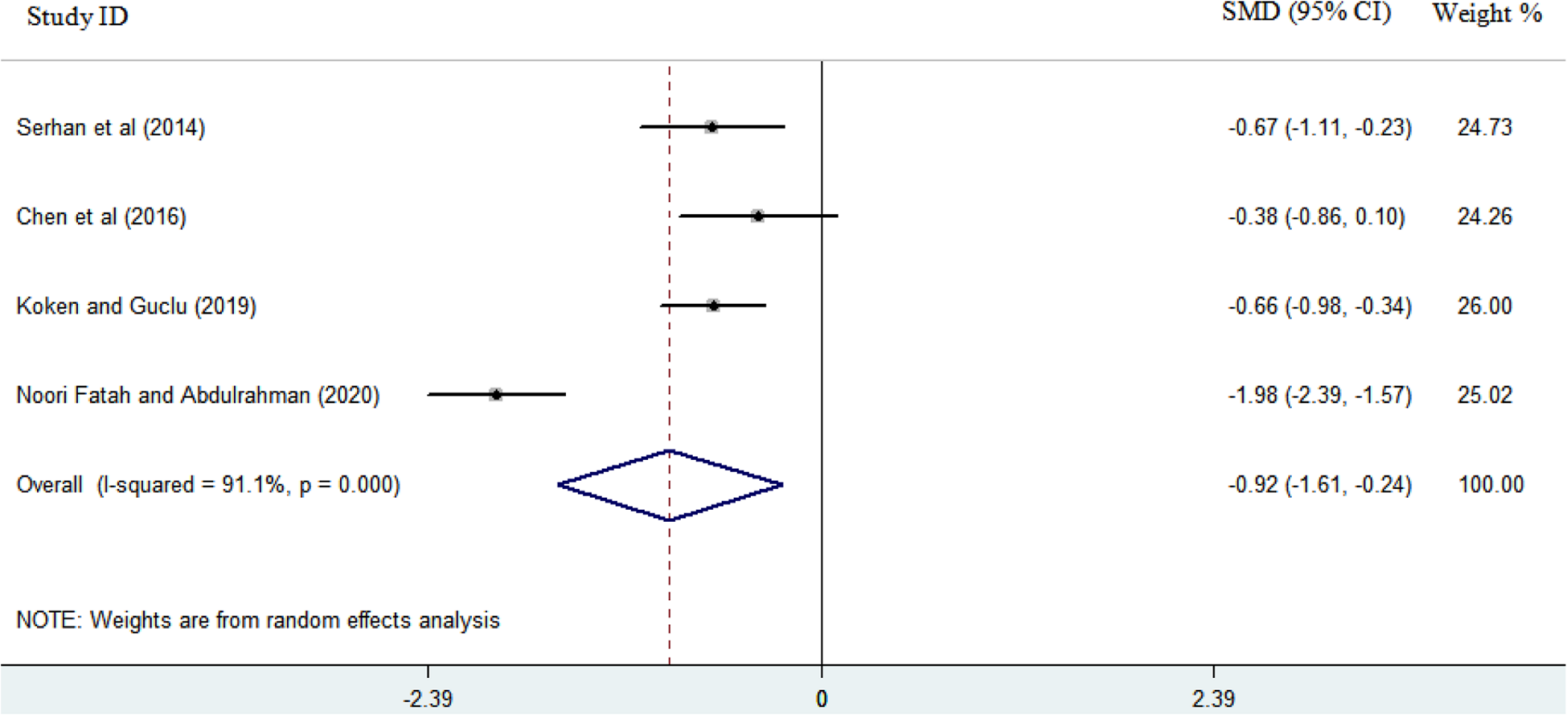
Forest plot showing the effects of TKA on sleep quality at time point of 3–6 months after surgery

Descriptive features of the studies included in this systematic review are shown in Table 1. As seen, except one case-control study, all other seven studies were designed as prospective or retrospective cohort. The results of the quality assessment of the studies, based on the NOS quality score, are shown in Table 1. All studies were of a moderate quality with a mean quality score of 7 (range 5–8). Of the eight studies that examined sleep quality in patients undergoing TKA, only four reported values for sleep quality based on the PSQI before and after surgery. According to the data provided in those studies and considering the time intervals for evaluating sleep quality after TKA, it is possible to perform a meta-analysis at two time points. The scores of sleep quality before surgery and at two time points afterward (near and far from the surgery) were extracted from the studies. The earlier time point for reporting mean PSQI score was considered 4–6 weeks after surgery, reported in only three studies. The later time point was considered 3–6 months after surgery, with four studies reporting patients' sleep quality. The random effect model was selected for analyzing the data. The standardized mean difference (SMD) change in sleep quality improvement was − 0.16 (− 1.05, 0.74) 4–6 weeks after surgery compared to preoperative values. The results of the meta-analysis (Fig. 2) show that the quality of sleep after TKA improved slightly 4–6 weeks after as compared to that before surgery. According to the Forest plot in Fig. 3, the quality of sleep improved 3–6 months after surgery by 0.92 as compared to the preoperative values, and the amount of SMD changes for this time point was -0.92 (− 1.61, − 0.024) compared to that before the surgery. Begg’s and Egger’s p values were respectively P > 0.602 and P = 0.818 for the meta-analysis performed on the sleep quality data at 4–6 weeks after surgery. These values at 3–6 months after surgery were determined as Begg (P > 0.497) and Egger (P = 0.936). Begg’s and Egger’s p values revealed no evidence of significant publication bias in both meta-analyses.

**Table 1:** Descriptive characteristics of the included studies

Authors	Year	Location	Type of study	No of participants	Sleep quality assessments tools	Check list	Total score
Cremeans-Smith et al. [20]	2006	Ohio	Prospective	110	PSQI	Ottawa/cohort	******
Mehmet Serhan et al. [24]	2014	Turkey	Prospective	42	PSQI	Ottawa/cohort	******
Herrero-Sánchez et al. [8]	2014	Spain	Case-control	24 case, 21 control	PSQI	Ottawa/case-control	********
Koken and Guclu [28]	2019	Turkey	Retrospective	80	PSQI	Ottawa/cohort	*******
Fatah and Abdulrahman [3]	2020	Iraq	Prospective Cohort	67	PSQI	Ottawa/cohort	*******
Luo et al. [29]	2019	China	Prospective Cohort	994	PSQI/ESS	Ottawa/cohort	*******
Manning et al. [9]	2017	Chicago	Prospective	62	ESS	Ottawa/cohort	*****
Chen et al. [5]	2016	Pennsylvania	Prospective	34	PSQI/ESS	Ottawa/cohort	*******

## Discussion

This study aimed to evaluate the prevalence and duration of sleep quality disorder after TKA. The meta-analysis results show the positive effects of TKA on sleep quality, especially at later time point after surgery. Undoubtedly, appropriate interventions are needed to improve patients’ sleep quality after such a major surgery. The reason for the deterioration of patients’ sleep quality in the first few days after surgery is related to the pain caused by the major surgery and the start of daily activities by the patient. PSQI scores are higher in the early postoperative period and are reported to decrease within 3 to 6 months after surgery, leading to further improvement of sleep quality.

Sarhan et al. reported that TKA facilitated patient’s onset of sleep, increased their sleep duration, and provided more effective sleep. Furthermore, the number of patients who woke up during sleep decreased and resulted in better daily functioning. After surgery, 78.6% of the patients reported improved sleep quality, and 97.6% reported improved pain scores. Although TKA improves both the visual analog score (VAS) and PSQI, the improvement observed in PSQI was independent from that of the pain [24].

Koken and Guclu reported that treatment of symptomatic osteoarthritis with TKA improves sleep quality in the long-term. They used spinal or epidural anesthesia in all operations. Pain scores based on VAS decreased 6 months after surgery compared to those before surgery and 6 weeks after (p < 0.05). PSQI scores before and after surgery were not associated with demographic variables or clinical data. Sleep quality decreased 6 weeks after surgery compared to that before surgery. The worst sleep quality, observed in the sixth week after surgery, pertained to the recovery period and pain due to the surgery. Moreover, a positive correlation was noted between VAS scores and the PSQI scores, indicating the effect of pain on sleep [28].

Noori et al. reported statistically insignificant association between PSQI score and variables of age, sex, BMI, a history of diabetes mellitus. In their research, all surgeries were performed under spinal anesthesia and the participants received postoperative opioids, acetaminophen, or NSAIDS for analgesia, with 76.1% of the patients having a catheter installed for epidural analgesia. They then assessed the impact of the postoperative analgesic regimen on sleep. During the follow-up, the pain gradually decreased. Sleep disturbances increased in the first month after surgery but gradually decreased. Sleep quality improved in 43% of patients, which continued until the end of follow-up (3 months after surgery). At the end of the follow-up period, a significant association was reported between the use of an epidural catheter for delivering analgesic agents and patients’ sleep quality. The use of this particular delivery method was shown to lead to better outcomes as compared with the patients who did not receive it. Moreover, patients with epidural catheter enjoyed a better early pain control than other patients [3].

In a study by Chen et al., all patients underwent TKA under spinal anesthesia and ambulated on the day of their surgery supervised by a physical therapist. During the postoperative hospitalization period, patients received oxycodone, oral acetaminophen and intravenous ketorolac. Results showed that patients undergoing knee replacement surgery should expect increased sleep disturbances in the short-run after the surgery (6 weeks), which will gradually decrease after three to 6 months. According to their study, compared to their pre-operative state, patients’ sleep quality deteriorated within 6 weeks after surgery (P = 0.03) but improved 3 and 6 months after surgery (P = 0.003). At all intervals after surgery, the amount of pain consistently decreased compared to its preoperative levels, and no change in the ESS was recorded over time. The VAS levels did not correlate with PSQI scores [5].

Herrero-Sánchez et al. examined the relationship between the demographic data of older adult patients undergoing surgery and variables of pain intensity, health-related quality of life, and sleep quality. Twenty-four patients who had surgery in the previous month and 21 controls enrolled in their study. Compared to the control group, individuals who underwent surgery had less physical activity, less social role, and more pain after surgery (P < 0.05). A group of patients who underwent surgery had a lower quality of life. The poorer functionality (compared to the control group) was reported based on the Western Ontario and McMaster Universities (WOMAC) index while poor sleep quality was reported based on the PSQI (P < 0.05). The high intensity of persistent postoperative pain was associated with lower functioning and quality of life as well as poor sleep quality [8].

A retrospective cohort study by Luo et al. examined the effects of preoperative sleep quality on postoperative outcomes. A preemptive analgesia using multimodal oral analgesic drugs (diclofenac and pregabalin) was used for pain control 1 day before surgery and repeated postoperative. Oral oxycodone or an intramuscular injection of parecoxib was administered if patients reported VAS greater than 4 or 6, respectively. PSQI scores were strongly associated with active and nocturnal pain scores and administration of analgesics, range of motion, and functional scores from the first to the third day after surgery. In the group who had knee replacement surgery, a significant correlation was observed between active pain scores and ESS scores 3 months after surgery. The use of analgesics after joint arthroplasty was highly related to PSQI scores. Therefore, the quality of sleep before surgery significantly affects postoperative pain, increases postoperative pain sensitivity, and leads to more analgesic administration [29].

Manning et al. reported that knee replacement surgery caused transient sleep disturbance in the early days after surgery, which improved over time. In the early postoperative period, i.e., about 4.7 ± 2.0 weeks, more sleep disturbances, including an increased duration of sleep (P = 0.006) and average sleep interruptions during the night (P = 0.002) were observed in patients, as compared to their preoperative state. Patients’ sleep quality improved at later postoperative time points (40.8 ± 19.5 weeks) and even exceeded the preoperative values [9].

Cremeans-Smith et al. examined the association between pain, sleep, and postoperative functional limitations in patients undergoing TKA. Their findings indicate a correlation between higher pain intensity and impaired sleep quality. Pain in 1 month after surgery was associated with sleep disorders p = 0.009 and the severity of arthritis p < 0.001. Sleep disorders were also significantly correlated with the severity of arthritis p = 0.002. This study emphasized that adequate sleep during postoperative recovery accelerated patient recovery [20].

The findings of this study can be used by both patients and orthopedic surgeons. Improving the quality of sleep in candidates for joint replacement surgery can improve the pain threshold, relieve postoperative pain, improve patient performance, reduce hospital stay, and reduce administration of painkillers. Since pain management methods are important in the early days after surgery, it is necessary for surgeons to control postoperative pain in order to improve the patients’ sleep quality. Patients should also expect pain and disturbed sleep in the early postoperative period, which will improve over time. Physicians should consider psychopharmacological interventions to improve sleep quality immediately after surgery. Moreover, due to the effect of disturbed preoperative sleep on the pain experience and pain sensitivity in patients, it is recommended that physicians evaluate patients’ sleep complaints and determine ways to optimize sleep quality before surgery.

One of the limitations of included studies is their small sample sizes. Another problem is their subjective tools such as surveys to assess patients’ sleep quality, while objective methods such as polysomnography provide more accurate results. Although the results of these studies showed the positive effects of orthopedic surgery on sleep quality, it is recommended that TKA be compared with other medical treatments. Further longitudinal studies are needed to assess postoperative sleep and other sleep-related factors. Finally, other limitations of the included studies were their non-random design and differences in their postoperative protocols/interventions.

## Conclusions

Although total knee arthroplasty is the only treatment for patients in the late stages of knee osteoarthritis, impaired sleep after surgery is very common among patients who undergo the surgery. The results show that TKA generally improves sleep quality although some studies report transient sleep disturbances. Certainly, postoperative medical interventions are necessary to control surgery-induced pain and to improve sleep quality. Since sleep affects patients’ recovery, surgeons should pay more attention to patients' sleep and consider relevant interventions.

## Data Availability

Not applicable.

## Abbreviations

ESS: Epworth Sleepiness Scale
NOS: Newcastle-Ottawa Scale
PSQI: Pittsburgh Sleep Quality Index
TKA: Total knee arthroplasty
VAS: Visual analog scale
WOMAC: Western Ontario and McMaster Universities
